# The Doctor and Politics

**Published:** 1895-11

**Authors:** 


THE DOCTORS AND POLITICS.—The East Texas Medical Society are trying their hands on a bill to "regulate the practice." At a recent meeting a committee of able members was appointed to draft a bill to regulate the practice, and they did so, presenting it at their last meeting, October 8th. But as there was not a quorum present, the secretary writes us, action was postponed three months. Mean time a copy of the bill has been submitted to the JOURNAL, and its opinion asked. The bill is very lengthy; too long, in fact. It provides for three distinct boards of medical examiners,—one of each "school," so called,—to be appointed out of a number of names to be submitted by the State society of the respective schools. The other provisions, in the main, are about like all bills which have heretofore been presented to the legislature, and ridiculed and kicked out.

Our East Texas friends surely have not forgotten that every year, since 1884, an effort has been made, by a committee from the State Medical Association, to secure the passage of a bill, and that some of the ablest men in the profession have tried their hands on it. The lamented Cupples; Wooten, McLaughlin, Tyner, many others, have, after carefully examining and comparing bills in successful operation in other States, drawn up bills year after year, and presented them in person. No matter what the form of bill, it has invariably been laughed at and pigeon holed, or, if pushed, has been defeated. The legislators say the doctors have no right to "regulate" anything. The JOURNAL has, for years, been begging getters-up of bills to abandon that form of petition; to ask for a bill to suppress quackery, or a bill to define the requisite qualifications of practicing physicians. We have been assured by senators that a bill of that kind will pass.

Now, let us reason together. We have seen that the legislators do not understand the situation, and do not appreciate the necessity for such legislation. The homeopaths, and the *vita*paths, and the faith healers, and all who would be cut off by such a bill as is needed, have effectually prejudiced successive legislators and made them believe there is a bug under the chip. We have just got to try another plan. Reverse the order; begin at the beginning. See our article elsewhere, the "Keynote to Success." Read it, ponder it and act on it. Let the State Medical Association, at its next meeting, appoint a member—or a doctor, in each county in the State, to organize the profession in his county; to get the doctors together and agree to personally see each candidate for House or Senate, and educate him; show him the burning necessity for a law to protect the people from such sharpers as the lordly Flower, who is now raiding the State, —and other conscienceless fellows, who, with just a smattering of knowledge, pretend to great skill; open their eyes to the fact that "quackery" is the heaviest tax our people have to bear; and as a condition to the physician's vote, make each candidate promise to support a suitable bill. Too many cooks spoil the broth, and we do not want the machinery too complicated. The shape of the bill, its provisions, can be considered later. What we want *now*, is to put the legislators in rapport with the doctors in the matter of suppressing quackery. The five thousand doctors in Texas are a power, able to make or mar the political fortune of any aspirant for legislative honors, if they will rightly go about it.

